# Highly Immunoresistant Acute Motor and Sensory Axonal Neuropathy With Concomitant Anti-NF155 Axonal Nodopathy: A Case Report

**DOI:** 10.7759/cureus.97181

**Published:** 2025-11-18

**Authors:** Nicholas M Riccione, Cassie N Chan

**Affiliations:** 1 Neurology, Health Science Center, The University of Texas at San Antonio, San Antonio, USA

**Keywords:** acute motor and sensory axonal neuropathy, chronic inflammatory demyelinating polyneuropathy, guillain barre, guillain-barré syndrome, neuromuscular, nodopathy

## Abstract

Acute motor and sensory axonal neuropathy (AMSAN) is a rare, severe subtype of Guillain-Barré syndrome (GBS) associated with a poorer prognosis and greater resistance to standard immunotherapies. While immunomodulatory therapies are the traditional mainstay therapies for GBS and its variants, as autoimmune-mediated processes, refractory cases can present unique challenges. In addition, chronic inflammatory demyelinating polyneuropathy (CIDP) is a known persistent variant of GBS with its own wide array of variations, including neurofascin IgG4-positive axonal nodopathy, associated with an antibody that provides an additional layer of immunoprotection. This condition has been found in previously documented case series to require non-traditional immunoregimens. We present a unique case of a highly refractory AMSAN with concomitant chronic axonal motor and sensory axonal nodopathy with high multimodal immunotherapy resistance. This case illustrates the importance of considering neurofascin IgG4 in refractory subacute axonal nodopathies and, consequently, the potential role of empiric rituximab, while also highlighting the need for further research into B-cell-depleting therapeutic alternatives in resistant cases.

## Introduction

Guillain-Barré syndrome (GBS) describes a broad category of acute (symptom severity peaking within four weeks), immune-mediated peripheral polyneuropathies often triggered by antecedent infection or, in some instances, vaccination [1]. The most prevalent GBS variant in the United States is acute inflammatory demyelinating polyneuropathy (AIDP), a diagnosis without an associated corresponding antibody yet identified, but known to target the myelin sheath [1]. In contrast, axonal GBS variants - acute motor axonal neuropathy (AMAN) and acute motor sensory axonal neuropathy (AMSAN) - are rare and generally more severe iterations, accounting for roughly 5% of GBS cases in Europe and North America, with higher percentages in Northern China and Japan [2]. 

When GBS symptoms persist beyond eight weeks, the diagnosis shifts to chronic inflammatory demyelinating polyneuropathy (CIDP). Compared to the monophasic nature of GBS, CIDP tends to present with a relapsing-remitting or progressive course. Like GBS, CIDP encompasses demyelinating and, less commonly, axonal subtypes, which can be distinguished via electrodiagnostic studies and specific autoantibodies.

Treatment of GBS and its variants relies on immunotherapies that neutralize circulating autoantibodies and/or inhibit complement system modulation, preventing further nerve damage. Plasmapheresis indiscriminately removes circulating autoantibodies, complement factors, cytokines, and other pro-inflammatory immune system components to reduce damage and hasten recovery [3]. On the other hand, intravenous immunoglobulin (IVIG) is theorized to neutralize autoantibodies and inhibit modulators of the complement system to reduce immune response [3]. Both modalities show equivalent efficacy in randomized trials. Similarly, both plasmapheresis and IVIG are used to treat CIDP for not only acute relapses but also as maintenance therapy. Additionally, in CIDP, corticosteroids and, for refractory cases, B-cell depleting agents, such as rituximab, can be utilized [4].

## Case presentation

**Figure 1:**
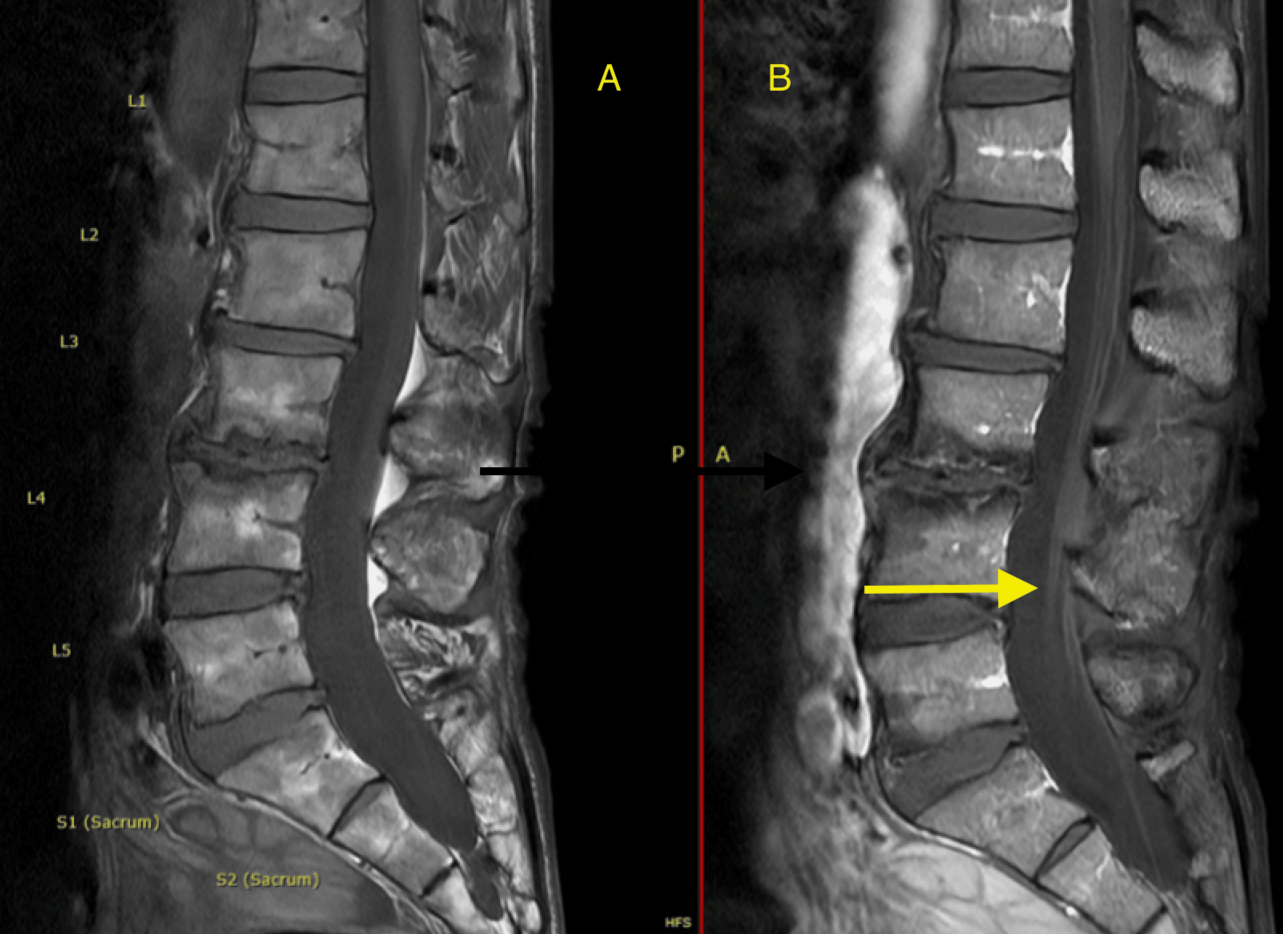
MRI lumbar spine with and without contrast. (A) Precontrast and (B) postcontrast image with evidence of cauda equina enhancement (yellow arrow).

A 69-year-old man with past medical history of idiopathic thrombocytopenia and hyperlipidemia, presented to the hospital for one month of progressive ascending bilateral lower extremity numbness extending to the mid-trunk, bowel and bladder urgency, and two falls five days into a treatment course with vancomycin for *Clostridium difficile* colitis. The neurologic exam on admission was significant for decreased vibration and light touch sensation to the mid-trunk, and absent lower extremity reflexes with full intact strength throughout. Respiratory measures (forced vital capacity and negative inspiratory force) were within normal limits. The initial ganglioside panel was GD1b-positive. MRI lumbar spine with and without contrast was significant for postcontrast cauda equina enhancement (Figures 1A, 1B).

Lumbar puncture was deferred due to high clinical suspicion of GBS. He was diagnosed with sensory-ataxic GBS and received intravenous immunoglobulin (IVIG) (2 g/kg) over five days. By discharge, his sensory exam was unchanged, and with full strength except for mild shoulder abduction weakness bilaterally.

Eight days later, he was readmitted for worsening weakness, new bowel/bladder urgency, and shortness of breath. On examination, cranial nerves were still intact, and deep tendon reflexes were decreased; however, sensation to light touch was absent in the lower extremities, and sensation from the shoulders to the hips was decreased. The motor strength exam was notable for significant diffuse weakness, with a general distal distribution greater than proximal (Table 1) [5].

**Table 1:** Follow-up muscle strength exam using MRC scale. Medical Research Council scale defined as "0= no muscle activation, 1=trace muscle activation, 2=muscle activation with gravity eliminated, 3=muscle activation against gravity, full range of motion, 4=muscle activation against some resistance, and 5=muscle activation against examiner’s full resistance." MRC: Medical Research Council

Motor strength	Right	Left
Shoulder abduction	2	2
Elbow flexion	2	2
Elbow extension	3	3
Wrist flexion	2	2
Wrist extension	2	2
Hand grip	2	2
Hip flexion	4	4
Knee flexion	4	4
Knee extension	4	4
Dorsiflexion	3	3
Plantar flexion	3	3

Subsequent cerebrospinal fluid analysis showed albuminocytologic dissociation. Repeat ganglioside testing remained GD1b-positive. Left upper and lower extremity motor nerve conduction study and right upper extremity sensory nerve conduction studies revealed reduced compound muscle action potentials (CMAPs), absent sensory nerve action potentials (SNAPs), with preserved conduction velocities consistent with AMSAN (Tables 2, 3). Electromyography (EMG) could not be performed due to significant electrical interference.

**Table 2:** Motor nerve conduction study performed with associated reference values. The significantly decreased compound muscle action potential amplitude with relatively preserved latency and conduction velocity indicates axon loss with relatively intact myelin. Reference values provided based on* *electromyography and neuromuscular disordersby Preston and Shapiro [6].

Variables	Distal latency (ms)	Compound muscle action potential (mV)	Conduction velocity (m/s)	F-wave latency (ms)
Left ulnar (abductor digiti minimi)	4.5 ms (reference value: <3.3 ms)	2 mV (reference value: >6 mV)	59 m/s (reference value: >49 m/s)	33.6 ms (reference value: <32 ms)
Left peroneal (extensor digitorum brevis)	8.35 ms (reference value: <6.5 ms)	0.3 mV (reference value: >2 mV)	43 m/s (reference value: >44 m/s)	Absent (reference value: <56 ms)
Left tibial (abductor hallucis brevis)	5.7 ms (reference value: <5.8 ms)	7.8 mV (reference value: >4 mV)	39.2 m/s (reference value: >41 m/s)	47.92 ms (reference value: <34 ms)

**Table 3:** Sensory nerve conduction performed with associated reference values. The lack of sensory responses indicates severe loss of large-diameter sensory nerve fibers, as most commonly seen in axonal degeneration. Reference values provided based on electromyography and neuromuscular disordersbyPreston and Shapiro [6]*.*

Variables	Distal latency (ms)	Sensory nerve action potential (μV)	Conduction velocity (m/s)
Right ulnar to digit V	No response (reference value: <3.1 ms)	No response (reference value: >17 μV)	No response (reference value: >50 m/s)
Right superficial radial	No response (reference value: <2.9 ms)	No response (reference value: >1 μV)	No response (reference value: >50 m/s)

He underwent five sessions of plasmapheresis, showing mild improvement in strength. On hospital day 17, he developed acute respiratory failure requiring bilevel positive airway pressure (BiPAP) and eventual intubation. A second round of IVIG was initiated but delayed due to pneumonia and septic shock. Five days of high-dose methylprednisolone (1000 mg) and a one-time dose of rituximab 1 g were then administered, but the patient progressed to quadriplegia. A tracheostomy and percutaneous endoscopic gastrostomy tube were placed. A Mayo chronic inflammatory demyelinating polyneuropathy (CIDP) panel sent before discharge eventually returned positive for NF155 IgG4 antibodies. The patient died one month later from a likely sequela related to his hospitalization. In total, the patient received two rounds of 2 g/kg of IVIG, five days of 1000 mg methylprednisolone, five sessions of plasmapheresis, and 1 g of rituximab.

## Discussion

Although the diagnosis of GBS and later AMSAN was confirmed via EMG/nerve conduction study (NCS), CSF albuminocytologic dissociation, and MRI of the lumbar spine, the patient had progressive neurologic decline despite multiple rounds of standard immunotherapy. This atypical resistance, coupled with a prolonged disease course, directed to a potential alternate or additional diagnosis, eventually identified as NF155 IgG4 antibody-positive chronic axonal nodopathy.

Historically, approximately 50% of GBS patients reach symptom nadir by two weeks, 80% by three weeks, and 90% by four weeks, with most patients beginning recovery at day 28 [2]. By day 200, 80% of patients achieve complete recovery [2]. Given the unexpected course observed in our patient, this rapid change strongly suggested the presence of a concomitant chronic axonal nodopathy, which was later confirmed to be NF155 IgG4 antibody-positive.

NF155 is a Schwann cell adhesion protein located at the paranodal myelin loops, essential for maintaining myelin-axon adhesion and signal propagation [7]. NF155 IgG4 antibodies target nodal/perinodal sites, disrupting nodal architecture without activating complement or triggering internalization, rendering IVIG and plasmapheresis largely ineffective [8]. As a result, B-cell therapies, such as rituximab, have shown promise in halting disease progression by targeting memory B-cells and plasma cells, preventing antibody production [8]. A recent multicenter retrospective observational study demonstrated that 77.3% of NF155 IgG4-positive patients responded positively to rituximab, with a significant improvement in the modified Rankin Scale [9]. In contrast, responses to IVIG, steroids, and plasmapheresis were much lower, at 13.1%, 27.8%, and 38.9%, respectively [9].

In our case, the resistance to IVIG, methylprednisolone, and plasmapheresis retrospectively is unsurprising given the eventual identification of NF155 IgG4 antibodies. However, the continued, stepwise progression despite multiple immunotherapy treatments, including rituximab, was unexpected [10]. The probability of such resistance, based on findings from the aforementioned multicenter study, was extremely low, cumulatively around 1.4%. Factoring in rituximab, this probability drops even further to 1.1% [9]. Though rituximab was administered, severe disability had already been incurred from circulating antibodies, so it is unclear if depleting B-cell population would have been effective at that time. Earlier rituximab administration could have potentially curtailed symptom progression, but this can only be conjectured. While no large-scale clinical trials have been conducted, several B-cell-depleting therapies, commonly used in multiple sclerosis, may offer promising alternatives for refractory cases.

## Conclusions

In conclusion, this case underscores the importance of early antibody testing in subacute, progressive axonal nodopathies that are refractory to standard immunotherapies. The patient’s resistance to multimodal immunotherapy pointed to a highly resistant variant later confirmed to be NF155 IgG4-positive nodopathy. Clinicians should consider empiric B-cell-targeted therapy, such as rituximab, early in the disease course to prevent irreversible damage. As resistance to rituximab may still occur, emerging immunotherapies used in other autoimmune conditions, such as anti-CD19 agents, warrant further investigation as potential alternatives in highly immunotherapy-refractory IgG4-related nodopathies.
